# Artificial Intelligence–Driven Serious Games in Health Care: Scoping Review

**DOI:** 10.2196/39840

**Published:** 2022-11-29

**Authors:** Alaa Abd-alrazaq, Israa Abuelezz, Asma Hassan, AlHasan AlSammarraie, Dari Alhuwail, Sara Irshaidat, Hashem Abu Serhan, Arfan Ahmed, Sadam Alabed Alrazak, Mowafa Househ

**Affiliations:** 1 AI Center for Precision Health Weill Cornell Medicine-Qatar Doha Qatar; 2 Division of Information and Computing Technology, College of Science and Engineering Hamad Bin Khalifa University Qatar Foundation Doha Qatar; 3 Information Science Department, College of Life Sciences, Kuwait University Kuwait Kuwait; 4 Health Informatics Unit, Dasman Diabetes Institute Kuwait Kuwait; 5 Department of Pediatrics, King Hussein Cancer Center Amman Jordan; 6 Department of Mechanical & Industrial Engineering, Faculty of Applied Science and Engineering, University of Toronto Toronto, ON Canada

**Keywords:** serious games, artificial intelligence, deep learning, machine learning, health care, digital health, eHealth, mobile phone

## Abstract

**Background:**

Artificial intelligence (AI)–driven serious games have been used in health care to offer a customizable and immersive experience. Summarizing the features of the current AI-driven serious games is very important to explore how they have been developed and used and their current state to plan on how to leverage them in the current and future health care needs.

**Objective:**

This study aimed to explore the features of AI-driven serious games in health care as reported by previous research.

**Methods:**

We conducted a scoping review to achieve the abovementioned objective. The most popular databases in the information technology and health fields (ie, MEDLINE, PsycInfo, Embase, CINAHL, IEEE Xplore, ACM Digital Library, and Google Scholar) were searched using keywords related to serious games and AI. Two reviewers independently performed the study selection process. Three reviewers independently extracted data from the included studies. A narrative approach was used for data synthesis.

**Results:**

The search process returned 1470 records. Of these 1470 records, 46 (31.29%) met all eligibility criteria. A total of 64 different serious games were found in the included studies. Motor impairment was the most common health condition targeted by these serious games. Serious games were used for rehabilitation in most of the studies. The most common genres of serious games were role-playing games, puzzle games, and platform games. Unity was the most prominent game engine used to develop serious games. PCs were the most common platform used to play serious games. The most common algorithm used in the included studies was support vector machine. The most common purposes of AI were the detection of disease and the evaluation of user performance. The size of the data set ranged from 36 to 795,600. The most common validation techniques used in the included studies were k-fold cross-validation and training-test split validation. Accuracy was the most commonly used metric for evaluating the performance of AI models.

**Conclusions:**

The last decade witnessed an increase in the development of AI-driven serious games for health care purposes, targeting various health conditions, and leveraging multiple AI algorithms; this rising trend is expected to continue for years to come. Although the evidence uncovered in this study shows promising applications of AI-driven serious games, larger and more rigorous, diverse, and robust studies may be needed to examine the efficacy and effectiveness of AI-driven serious games in different populations with different health conditions.

## Introduction

### Background

Since its establishment in the 21st century, video games have experienced a boom and become an ever-growing global industry [1]. In recent years, there has been a rapid increase in the accessibility and ubiquity of handheld computers and smart devices (ie, tablets, wearables, and smartphones) as well as major advances in the underlying technology and capability of commercial video game consoles [2], thereby providing a plethora of opportunities to leverage video games for many purposes. Video games used for purposes other than entertainment (eg, education, training, research, rehabilitation, and advertising) are called serious games [3].

In health care, serious games have been used for many purposes such as screening, diagnosing, education, prevention, and rehabilitation [4,5]. For example, serious games have shown promising results in improving health education [6]; acute pain management [7]; cognitive functions (eg, global cognition [8], memory [9], executive functions [10], and processing speed [11]); mental health disorders (eg, depression [12] and anxiety [13]); and functional, motor, and sensory functions [14]. Furthermore, serious games have the potential to diagnose and screen many diseases such as mild cognitive impairment [15], developmental dyslexia [16], and attention-deficit/hyperactivity disorder [17].

Serious games rely on the concept of gamification, which involves the “use of game design elements within non-game contexts” [18] through the structure, design, and methodology of games [19]. According to evidence, gamification typically relies on three elements: (1) game dynamics, including the behaviors, interactions, and experience of the player; (2) pedagogical or instructional design of the game; and (3) the mechanics (ie, procedures and rules) of the game [20]. Typically, gamification relies on the use of points, badges, leader boards, or timed performance [21,22].

Serious games exist in several formats depending on their therapeutic modality such as (1) exergames, which are video games that require physical activity to be played [23]; (2) computerized cognitive behavioral therapy games, which provide the player with structured approaches to address and recognize negative thinking and beliefs [24]; (3) cognitive training games that target improving or maintaining the player’s cognitive abilities, including executive functions, memory, and learning [25]; or (4) biofeedback games that use electrical sensors attached to the player to receive information about the player’s physiological state and in turn influence some of the player’s body functions (eg, heart rate) [26,27].

Experts suggest that artificial intelligence (AI) is positioned to broadly reshape health care and the practice of medicine [28]. Coined by John McCarthy in a lecture at Dartmouth College in 1956 [29], AI is a branch of computer science that involves the development of methods, techniques, and systems that intelligently handle and analyze complex data sets and information. In recent years, AI models have played an increasingly central role in medical research and clinical practice through several applications including personalized screening, diagnosis, prognosis, monitoring, risk modeling, drug discovery, and prediction of response to therapy [30,31].

AI-driven serious games, which are video games combined with AI used for purposes other than entertainment, for health can offer a customizable and immersive experience that adjusts its speed and difficulty, for example, based on the player’s performance [1]. Through the use of AI algorithms, serious games can monitor the performance of players in real time [32]. For example, using data mining, serious games that leverage AI can evaluate players’ behaviors, mood, and personality while playing a serious game [33]. In addition, AI-driven serious games that use data mining techniques can improve players’ knowledge, skills, and training progress through the analysis of the data collected playing the game [34,35].

### Research Problem and Aim

Several studies have been conducted on AI-driven serious games in health care. Summarizing the features of the current AI-driven serious games is very important to explore how they have been developed and used and their current state to plan on how to leverage them in the current and future health care needs. Previous reviews did not focus on AI-driven serious games [36] and focused on a specific disease rather than health care in general [8-13]. Therefore, this review aimed to explore the features of AI-driven serious games in health care as reported by previous studies. Thus, this review focused on both AI and serious games together rather than serious games alone. Furthermore, our review is more comprehensive than other reviews, as it targeted serious games for any health condition rather than targeting a specific health condition.

## Methods

### Overview

To achieve the abovementioned objective, we conducted a scoping review in line with the guidelines of PRISMA-ScR (Preferred Reporting Items for Systematic Reviews and Meta-Analyses extension for Scoping Reviews) [37]. Multimedia Appendix 1 shows the PRISMA-ScR checklist for this review. The methods used in this review are described in detail in the following subsections.

### Search Strategy

#### Search Sources

The following databases were searched on January 12, 2022: MEDLINE (via Ovid), PsycInfo (via Ovid), Embase (via Ovid), CINAHL (via EBSCO), IEEE Xplore, ACM Digital Library, and Google Scholar. In the case of Google Scholar, only the first 100 publications were considered because it retrieved a massive number of publications, and we found that the results quickly lost relevance and applicability beyond the first 100 hits. To identify further studies, we screened the reference lists of the included studies and relevant reviews (ie, backward reference list checking), and we checked the studies that cited the included studies (ie, forward reference list checking) [38].

#### Search Terms

The search query in this review was developed by consulting 3 experts in digital health and by checking the search queries used in previous reviews within this area. The developed search query is composed of AI-related terms (eg, AI, machine learning, and deep learning) and serious games–related terms (eg, serious games and exergames). The search query used to search each of the 8 databases is shown in Multimedia Appendix 2.

### Study Eligibility Criteria

In this review, we included only studies that focused on AI-driven serious games used for any purpose in health care (eg, diagnosis, rehabilitation, prognosis, quantification, screening, and forecasting). We focused only on serious games that are played on any digital platform (eg, computers, consoles, mobile phones, and handheld devices), whereas nondigital games and those used in other fields (eg, education) were excluded. We also focused on serious games provided to health consumers (patients or healthy people) rather than health care providers or caregivers. We excluded studies that provided an overview or proposal for AI-driven serious games. This review included only empirical studies written in English. Although we included peer-reviewed articles, dissertations, conference proceedings, and preprints, we excluded reviews, conference abstracts, proposals, editorials, and commentaries. We did not apply any restrictions on the year of publication, country of publication, study design, population, and outcomes.

### Study Selection

The study selection process in this review consisted of three steps: (1) removing duplicates from all retrieved studies using EndNote, (2) screening titles and abstracts of the remaining publications, and (3) reading the entire text of the studies included in the previous step. In the full-text screening, we read the paper from title to conclusion in addition to the supplementary materials. Two reviewers independently performed the study selection process. Disagreements between the reviewers in the second and third steps were resolved by consulting 2 other reviewers. Cohen κ was calculated to measure the reviewer’s agreement [39], and it was 0.81 for title and abstract screening and 0.86 for full-text reading.

### Data Extraction

Multimedia Appendix 3 displays the data extraction form used in this review, which was pilot-tested using 5 included studies. Three reviewers independently used Microsoft Excel to extract data related to the characteristics of the included studies, serious games, and AI techniques. Any disagreement between the reviewers was resolved through discussion.

### Data Synthesis

A narrative approach was used to synthesize data extracted from the included publications. Specifically, we began by describing the features of serious games used in the included studies in terms of their name, target condition, purpose, therapeutic modality, connectivity, interface, genre, types, and platform. Then, we described the features of the AI techniques used in the included studies in terms of their purposes, AI algorithms, type of data, size of the data set, type of validation, and performance. We used Microsoft Excel to manage data synthesis.

## Results

### Search Results

**Figure 1:**
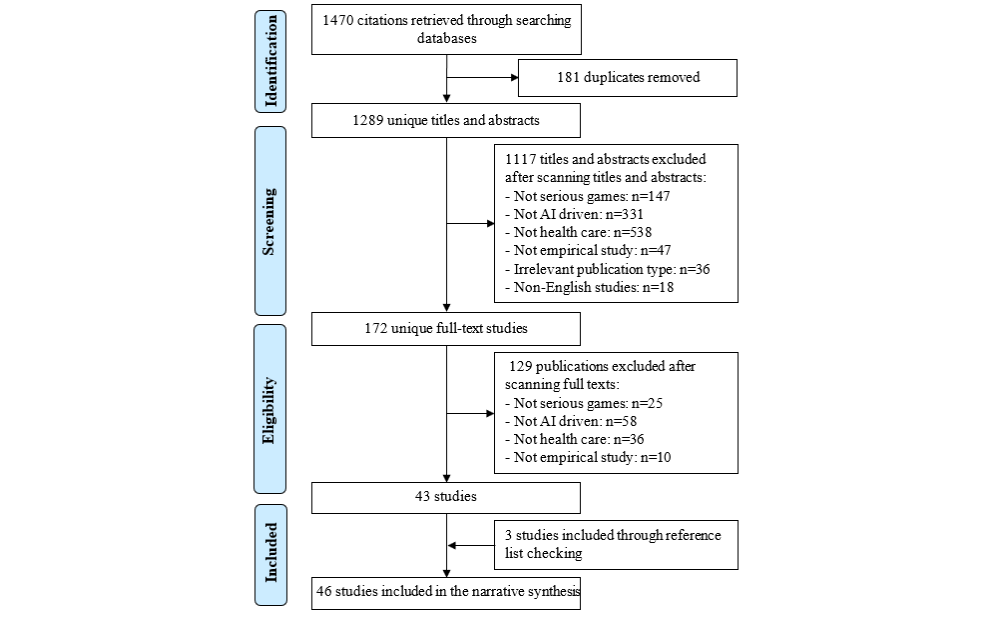
Flowchart of the study selection process. AI: artificial intelligence.

The total number of publications retrieved by searching the predefined databases was 1470 (Figure 1). We removed 181 duplicates from those publications. Checking the titles and abstracts of the remainders led to the exclusion of 1117 publications due to several reasons, as shown in Figure 1. After checking the full text of the remaining 172 publications, 129 were excluded for several reasons, as shown in Figure 1. We identified 3 additional studies using backward and forward reference list checking. Accordingly, the final number of included studies was 46 [40-85].

### Characteristics of the Included Studies

The included studies were published between 2010 and 2021. The years wherein the largest number of included studies were published were 2018 (8/46, 2%) and 2019 (7/46, 15%). The included studies were conducted in 30 different countries. The countries that published the largest number of studies were the United States and Spain (5/46, 11%). The included studies were published in peer-reviewed journals (26/46, 57%) or in conference proceedings (20/46, 44%). Multimedia Appendix 4 [40-85] presents the characteristics of each included study.

### Characteristics of the Serious Games

A total of 64 different serious games were found in the included studies (6 studies used >1 serious game). Of the 64 games, 16 (25%) were not given a specific name. Serious games were used for 20 health conditions. Motor impairment was the most common health condition targeted by the serious games in the included studies (18/46, 39%), followed by attention deficit hyperactivity disorder (4/46, 9%). Serious games were used for 5 purposes: rehabilitation (29/46, 63%), detection of diseases or disorders (10/46, 22%), health and wellness (5/46, 11%), education (2/46, 4%), and the prediction of players’ characteristics (1/46, 2%). The therapeutic modalities in the 29 rehabilitation games were exercise (19/29, 66%), cognitive training (6/29, 21%), and biofeedback (5/29, 17%). The interface of the serious games was 2D in 19 studies, 3D in 23, and 2D and 3D in 4 studies. Serious games in the included studies could be played by a single player (45/46, 98%) or multiplayer (1/46, 2%). Serious games were connected to an internet or network connection (ie, web-based games) in 8 studies, whereas they were played on stand-alone devices (offline games) in 38 studies. There were 10 genres of serious games in the included studies, but the most common genres were role-playing games (14/46, 30%), puzzle games (11/46, 24%), platformer games (10/46, 22%), and sports games (8/46, 17%). The games were designed with a *serious* purpose from the beginning (designed serious games) in 42 studies, whereas they were usual video games that were modified to be used for a serious purpose (modified serious games) in 4 studies. The game engine used to develop serious games was reported in 38 studies. Unity was the most prominent game engine used in these studies (19/38, 50%). We identified 6 platforms used to play the serious games: PCs (30/46, 65%), mobile devices (eg, mobile phones and tablets; 10/46, 22%), virtual reality headset (4/46, 9%), treadmill (1/46, 2%), Sifteo cubes (1/46, 2%), and a single-board computer (Raspberry Pi; 1/46, 2%). The serious games were connected with other devices in 36 studies: nonwearable sensors (18/46, 39%), wearable sensors (15/46, 33%), wearable devices (7/46, 15%), web camera (7/46, 15%), robotic device (3/46, 7%), microphone (3/46, 7%), controllers (2/46, 4%), smartphone (1/46, 2%), monitor (1/46, 2%), speakers (1/46, 2%), and single-board computer (tiny PC; 1/46, 2%). Multimedia Appendix 5 [40-85] shows characteristics of the serious games in the included studies. 

### Characteristics of the AI Techniques

The included studies used algorithms to solve classification problems (41/46, 89%), regression problems (5/46, 11%), and clustering problems (2/46, 4%). Algorithms embedded in serious games were reported in 40 studies, whereas the remaining studies did not report the algorithms used. These studies used 27 different algorithms for serious games. The most common algorithm used in the included studies was support vector machine (14/46, 30%), followed by convolutional neural network (7/46, 15%), artificial neural networks (7/46, 15%), and random forest (7/46, 15%). AI algorithms in the included studies were used for 9 different purposes: detection of disease (13/46, 28%), evaluation of user performance (13/46, 28%), adaptation of difficulty level (7/46, 15%), recognition of gestures (7/46, 15%), recognition of biosignals (5/46, 11%), supporting users to play (3/46, 7%), classification of activity (2/46, 4%), recognition of voice (2/46, 4%), and prediction of user characteristics (1/46, 2%). Multimedia Appendix 6 [40-85] exhibits characteristics of AI techniques leveraged by serious games in the included studies.

The AI models in the included studies were developed using the following types of data: kinematic data (22/46, 48%), gameplay data (21/46, 46%), biosignal data (11/46, 24%), demographic data (3/46, 7%), speech data (2/46, 4%), clinical data (1/46, 2%), and laboratory data (1/46, 2%). Data used for developing the models were collected from samples ranging from 3 to 150, as reported in 36 studies. The mean sample size was approximately 36 (SD 39.3). The data set size was reported in 24 studies and ranged from 36 to 795,600, with an average of approximately 52,124 (SD 161,862.2). The AI models in the included studies were validated using 4 techniques: k-fold cross-validation (13/46, 28%), training-test split validation (13/46, 28%), leave-one-out cross-validation (7/46, 15%), and moving-window cross-validation (1/46, 2%). The performance of the AI models was evaluated in 32 studies using 11 different metrics: accuracy (26/46, 57%), sensitivity (13/46, 28%), *F*_1_-score (9/46, 20%), precision (7/46, 15%), specificity (6/46, 13%), negative predictive value (3/46, 7%), area under the curve (3/46, 7%), root mean square error (1/46, 2%), normalized root mean square error (1/46, 2%), kappa (1/46, 2%), and Mathew correlation coefficient (1/46, 2%).

## Discussion

### Principal Findings

This study summarized the evidence about the features of AI-driven serious games in health care as reported by previous research. The 64 AI-driven serious games uncovered by this study targeted 20 different health conditions, were built for various purposes, and leveraged several therapeutic modalities through the use of multiple AI algorithms. The evidence uncovered in this review points to a rising trend in the use of AI-driven serious games in health care in recent years. The findings reported in this review were consistent with other recent evidence. Although a review by Frutos-Pascual and Zapirain [1] did not solely focus on AI-based serious games for health care purposes, its findings related to AI-based serious games were consistent with our findings with respect to their potential application for health care purposes, AI algorithms used, and the platforms used; there is also agreement about the need for improved testing methodologies to ensure efficacy.

Although the studies included in this review were conducted across the globe, many were conducted in 1 country. Therefore, the evidence remains scarce with respect to the compatibility of AI-driven serious games with the sociocultural practices of consumers playing them. Literature indicates that understanding a community’s sociocultural practices can significantly contribute toward designing and building reliable serious games; hence, more studies in the reported countries, as well as others, are needed [86].

Most of the AI-driven serious games reported in the studies examined in this review were heavily focused on the interventional therapeutics and the detection of diseases or disorders compared with prevention (ie, health and wellness or education). Given the alarmingly rising rates of noncommunicable diseases globally (eg, diabetes and cardiovascular diseases) [87], it is imperative to invest more efforts in developing more AI-driven serious games that focus on prevention and not only treatment and therapy because of the potential of serious games in providing systematic and sustainable means of preventing or delaying the onset of such noncommunicable diseases [5,87,88].

A recent study that developed a smartphone-based serious game that teaches self-management to children aged 8 to 14 years with type 1 diabetes reported that although the developed prototype of the serious game was perceived as useful and engaging by participants, it was not adaptable to players’ knowledge level and provided “information [that] was too basic for participants” [89]. This presents a great opportunity for developing AI-driven serious games that adapt to players’ abilities and knowledge level [1,90,91], making them more engaging and meaningful.

The studies examined in this review that reported the game engine used to develop their AI-driven serious game predominantly used the proprietary game engine *Unity* (19/38, 50%). There is room for further development of AI-driven serious games on open-source platforms [92], which can make their development collaborative, modular, and modifiable [93]. In addition, half of the studies examined in this review required players to play the AI-driven serious game on a PC. This goes against the fast-paced adoption and ubiquity of smart devices, such as smartphones and tablets.

Although only 4 studies reported the use of virtual reality headsets, we speculate that this number will rise in the years to come with the hype of *metaverses* and availability as well as affordability of these headsets. This progression comes naturally with the increasing adoption of connected devices, including wearable and nonwearable sensors, as part of the AI-driven serious game. With this in mind, we project that AI-driven serious games will be more adaptable in an unobtrusive and affordable manner [94].

This review found that 3D serious games were slightly more common than 2D serious games, which is in line with the findings of a previous review [36]. This can be attributed to the fact that 3D games are more immersive and attractive to players. Although 4 studies used both 2D and 3D serious games, none of the serious games in these 4 studies had multimodal interfaces. More precisely, each study included >1 serious game, and the interface of each game was either 2D or 3D rather than multimodal (2D and 3D). It is worth noting that none of these studies compared the effectiveness of a 2D serious game with a 3D serious game.

### Practical and Research Implications

#### Practical Implications

Summarizing the features of the current AI-driven serious games helped us explore how they have been developed and used and their current state, and this will help us plan on how to leverage them in the current and future health care needs. Only 10 studies in this review used smart mobile devices (ie, tablets and smartphones). The ubiquity of smart mobile devices, coupled with their increasing capabilities, affordability, and accessibility, makes them more appealing for future applications of AI-driven serious games, and smart mobile devices are certainly more pervasive compared with personal computers and gaming consoles [8]. Estimates of global mobile devices and mobile users are reported to be 15 billion and 7.1 billion, respectively [95].

There is a need to consider the sociocultural context and player demographics when designing and developing AI-driven serious games. In addition, involving multiple stakeholders, including the targeted audience (ie, consumers or patients), is fundamental to the success of an AI-driven serious game [96,97].

#### Research Implications

Of the 64 studies examined in this review, 14 (22%) did not report the performance of the AI models used in the serious games. The evidence uncovered in this study demonstrates a promising potential for leveraging AI-driven serious games for health care purposes, which in turn can inform future research efforts by demonstrating the status quo of research in this domain. With the increasing adoption of AI in medical software and the development of serious games, and considering that AI models may not be fully explainable at times, it becomes imperative to rigorously test and report the performance of the models, especially in high-stakes use cases such as missing a diagnosis of disease [98].

The studies included in this review had sample sizes ranging from 3 to 150, with many of them in the lower range. More evidence and research are needed on larger sample sizes to determine the generalizability of the findings and the impact of AI-driven serious games. It is also essential to examine the efficacy and effectiveness of AI-driven serious games in different populations with different health conditions. Although many of the studies examined in this review reported the data set size used, numerous studies did not; therefore, we urge researchers to not only report the data set size but also increase it to ensure adequate performance of AI-driven serious games for health care purposes [99]. In addition, more research, including randomized control trials and systematic reviews, may be needed to examine the efficacy and effectiveness of AI-driven serious games in different populations with different health conditions.

### Strengths and Limitations

#### Strengths

To the best of our knowledge, this is the first review of AI-driven serious games in health care. Only 1 previous review focused on serious games in health care; however, it did not focus on AI-driven serious games. Furthermore, this review can be considered the most comprehensive review in this area, given that it focused on all AI-driven serious games in health care regardless of their target health condition, therapeutic modality, game interface, number of players, connectivity, genre, type, game engine, platform, AI techniques, data types, sample size, data set size, and validation methods.

Bias resulting from the study selection was minimal in the review because the 2 reviewers independently performed the study selection process, and any disagreements between them were resolved by consulting 2 other reviewers. Furthermore, bias resulting from data extraction is not a concern in this review, as 3 reviewers independently extracted data from the included studies, and any disagreement between them was resolved through discussion. Bias resulting from missing papers is minimal, given that we sought to retrieve as many relevant studies as possible by searching the most popular databases in the information technology and health fields using a well-developed search query and by conducting backward and forward reference list checking.

#### Limitations

This review may have missed some relevant studies, given that we excluded proposals of AI-driven serious games (ie, a conceptual framework of a serious game), studies written in a language other than English, and studies focused on AI-driven serious games for health care providers and caregivers. Furthermore, it is likely that we missed some relevant papers, given that we did not search on Scopus and Web of Science. Therefore, it is likely that we missed other applications and features of AI-driven serious games. It was difficult to synthesize data related to the performance of AI-driven serious games for the following reasons: (1) the included studies had considerable heterogeneity in terms of game features (eg, target health condition, therapeutic modality, game interface, genre, and type), AI techniques (eg, their purpose, data type, and validation methods), and performance metrics and (2) conclusions drawn from such synthesis of games’ performance may be misleading because the risk of bias in the included studies was not assessed in this review. Therefore, this review could not comment on the performance of AI-driven serious games.

### Conclusions

The last decade witnessed an increase in the development of AI-driven serious games for health care purposes, and this rising trend is expected to continue for years to come. In this review, the 64 AI-driven serious games had varying data set sizes, ranging from only 36 to 795,000; these games reported targeting various health conditions, with motor impairment being the most common, and were mainly used for several therapeutic modalities, with rehabilitation being the most reported. In addition, these AI-driven serious games reported leveraging multiple AI algorithms, with support vector machines being the most used. Although the evidence uncovered in this study shows promising applications of AI-driven serious games, and considering the rise and rapid advances in AI and its pervasive use in serious games in the last decade, larger, more rigorous, diverse, and robust studies may be needed to examine the efficacy and effectiveness of AI-driven serious games in different populations with different health conditions. AI-driven serious games are expected to be a popular source to inspire the development and design of nearly realistic health-related and preventive interventions. Further evidence is necessary to determine their efficacy and performance.

## Appendix

Multimedia Appendix 1 PRISMA-ScR (Preferred Reporting Items for Systematic Reviews and Meta-Analyses extension for Scoping Reviews) checklist.

Multimedia Appendix 2 Search strategy.

Multimedia Appendix 3 Data extraction form.

Multimedia Appendix 4 Characteristics of each included study.

Multimedia Appendix 5 Characteristics of the serious games in the included studies.

Multimedia Appendix 6 Characteristics of artificial intelligence techniques leveraged by serious games in the included studies.

## Abbreviations

AI: artificial intelligence
PRISMA-ScR: Preferred Reporting Items for Systematic Reviews and Meta-Analyses extension for Scoping Reviews
