# Supplementary material for: Artificial Intelligence–Driven Serious Games in Health Care: Scoping Review
Source: JMIR Serious Games. 2022 Nov 29;10(4):e39840. doi: 10.2196/39840 (PMC9748798; doi:10.2196/39840)
Supplement: Multimedia Appendix 2 [file games_v10i4e39840_app2.docx]

**Multimedia Appendix 2: Search strategy**

Database(s): **Ovid MEDLINE(R) ALL**1946 to January 12, 2022
Search Strategy:

| **#** | **Searches** | **Results** |
| --- | --- | --- |
| 1 | exp Artificial Intelligence/ | 122547 |
| 2 | Artificial Intelligence.tw. | 13590 |
| 3 | exp Machine Learning/ | 32331 |
| 4 | Machine Learning.tw. | 43391 |
| 5 | exp Deep Learning/ | 6959 |
| 6 | Deep Learning.tw. | 18610 |
| 7 | exp Neural Networks, Computer/ | 37261 |
| 8 | "Decision tree".tw. | 8807 |
| 9 | "K-Nearest Neighbor*".tw. | 3265 |
| 10 | "Support vector machine*".tw. | 18337 |
| 11 | "Recurrent neural network*".tw. | 2654 |
| 12 | "convolutional neural network*".tw. | 11442 |
| 13 | "Artificial neural network*".tw. | 12819 |
| 14 | "Naïve Bayes".tw. | 2 |
| 15 | "Naive Bayes".tw. | 2019 |
| 16 | "Fuzzy Logic".tw. | 2007 |
| 17 | "logistic regression".tw. | 319694 |
| 18 | "K-Means".tw. | 4903 |
| 19 | "Random Forest".tw. | 10364 |
| 20 | "Long Short-Term Memory Networks".tw. | 104 |
| 21 | exp Video Games/ | 6229 |
| 22 | "serious gam*".tw. | 849 |
| 23 | "game-based".tw. | 772 |
| 24 | "videogam*".tw. | 826 |
| 25 | "video game*".tw. | 3681 |
| 26 | gamification.tw. | 693 |
| 27 | gamified.tw. | 367 |
| 28 | exergam*.tw. | 784 |
| 29 | "Applied game*".tw. | 23 |
| 30 | "virtual reality game*".tw. | 125 |
| 31 | "Virtual reality-based game*".tw. | 3 |
| 32 | "augmented reality game*".tw. | 35 |
| 33 | "augmented reality-based game*".tw. | 0 |
| 34 | 1 or 2 or 3 or 4 or 5 or 6 or 7 or 8 or 9 or 10 or 11 or 12 or 13 or 14 or 15 or 16 or 17 or 18 or 19 or 20 | 504940 |
| 35 | 21 or 22 or 23 or 24 or 25 or 26 or 27 or 28 or 29 or 30 or 31 or 32 or 33 | 10317 |
| 36 | 34 and 35 | 565 |
| 37 | limit 36 to yr="2010 -Current" | 510 |
| 38 | limit 37 to english language | 504 |
| 39 | limit 38 to humans | 412 |

Database(s): **Embase**1996 to 2022 Week 2
Search Strategy:

| **#** | **Searches** | **Results** |
| --- | --- | --- |
| 1 | exp Artificial Intelligence/ | 50604 |
| 2 | Artificial Intelligence.tw. | 16446 |
| 3 | exp Machine Learning/ | 267821 |
| 4 | Machine Learning.tw. | 52309 |
| 5 | exp Deep Learning/ | 17995 |
| 6 | Deep Learning.tw. | 21938 |
| 7 | exp Neural Networks, Computer/ | 58461 |
| 8 | "Decision tree".tw. | 12600 |
| 9 | "K-Nearest Neighbor*".tw. | 3923 |
| 10 | "Support vector machine*".tw. | 22546 |
| 11 | "Recurrent neural network*".tw. | 2978 |
| 12 | "convolutional neural network*".tw. | 13819 |
| 13 | "Artificial neural network*".tw. | 15107 |
| 14 | "Naïve Bayes".tw. | 17 |
| 15 | "Naive Bayes".tw. | 2518 |
| 16 | "Fuzzy Logic".tw. | 2373 |
| 17 | "logistic regression".tw. | 459934 |
| 18 | "K-Means".tw. | 6668 |
| 19 | "Random Forest".tw. | 13386 |
| 20 | "Long Short-Term Memory Networks".tw. | 113 |
| 21 | exp Video Games/ | 4465 |
| 22 | "serious gam*".tw. | 962 |
| 23 | "game-based".tw. | 844 |
| 24 | "videogam*".tw. | 995 |
| 25 | "video game*".tw. | 4451 |
| 26 | gamification.tw. | 754 |
| 27 | gamified.tw. | 355 |
| 28 | exergam*.tw. | 830 |
| 29 | "Applied game*".tw. | 22 |
| 30 | "virtual reality game*".tw. | 167 |
| 31 | "Virtual reality-based game*".tw. | 4 |
| 32 | "augmented reality game*".tw. | 30 |
| 33 | "augmented reality-based game*".tw. | 0 |
| 34 | 1 or 2 or 3 or 4 or 5 or 6 or 7 or 8 or 9 or 10 or 11 or 12 or 13 or 14 or 15 or 16 or 17 or 18 or 19 or 20 | 766217 |
| 35 | 21 or 22 or 23 or 24 or 25 or 26 or 27 or 28 or 29 or 30 or 31 or 32 or 33 | 10125 |
| 36 | 34 and 35 | 536 |
| 37 | limit 36 to yr="2010 -Current" | 504 |
| 38 | limit 37 to english language | 497 |
| 39 | limit 38 to humans | 463 |
| 40 | limit 39 to exclude medline journals | 64 |

Database(s): **APA PsycInfo**2002 to January Week 2 2022
Search Strategy:

| **#** | **Searches** | **Results** |
| --- | --- | --- |
| 1 | exp Artificial Intelligence/ | 21039 |
| 2 | Artificial Intelligence.tw. | 4102 |
| 3 | exp Machine Learning/ | 10816 |
| 4 | Machine Learning.tw. | 8154 |
| 5 | exp Deep Learning/ | 0 |
| 6 | Deep Learning.tw. | 1937 |
| 7 | exp Neural Networks, Computer/ | 0 |
| 8 | "Decision tree".tw. | 1193 |
| 9 | "K-Nearest Neighbor*".tw. | 402 |
| 10 | "Support vector machine*".tw. | 2837 |
| 11 | "Recurrent neural network*".tw. | 824 |
| 12 | "convolutional neural network*".tw. | 842 |
| 13 | "Artificial neural network*".tw. | 1796 |
| 14 | "Naïve Bayes".tw. | 1 |
| 15 | "Naive Bayes".tw. | 400 |
| 16 | "Fuzzy Logic".tw. | 658 |
| 17 | "logistic regression".tw. | 55132 |
| 18 | "K-Means".tw. | 1262 |
| 19 | "Random Forest".tw. | 773 |
| 20 | "Long Short-Term Memory Networks".tw. | 21 |
| 21 | exp Video Games/ | 7666 |
| 22 | "serious gam*".tw. | 961 |
| 23 | "game-based".tw. | 1592 |
| 24 | "videogam*".tw. | 1022 |
| 25 | "video game*".tw. | 5439 |
| 26 | gamification.tw. | 778 |
| 27 | gamified.tw. | 381 |
| 28 | exergam*.tw. | 386 |
| 29 | "Applied game*".tw. | 19 |
| 30 | "virtual reality game*".tw. | 69 |
| 31 | "Virtual reality-based game*".tw. | 1 |
| 32 | "augmented reality game*".tw. | 51 |
| 33 | "augmented reality-based game*".tw. | 0 |
| 34 | 1 or 2 or 3 or 4 or 5 or 6 or 7 or 8 or 9 or 10 or 11 or 12 or 13 or 14 or 15 or 16 or 17 or 18 or 19 or 20 | 84892 |
| 35 | 21 or 22 or 23 or 24 or 25 or 26 or 27 or 28 or 29 or 30 or 31 or 32 or 33 | 11866 |
| 36 | 34 and 35 | 338 |
| 37 | limit 36 to yr="2010 -Current" | 304 |
| 38 | limit 37 to english language | 291 |
| 39 | limit 38 to humans [Limit not valid in APA PsycInfo; records were retained] | 291 |

Database(s): **CINAHL**Sunday, January 12, 2022
Search Strategy:

| **#** | **Searches** | **Results** |
| --- | --- | --- |
| S1 | MW "Artificial Intelligence" OR TI "Artificial Intelligence" OR AB "Artificial Intelligence" | 7,689 |
| S2 | MW "Machine Learning" OR TI "Machine Learning" OR AB "Machine Learning" | 8,446 |
| S3 | MW "Deep Learning" OR TI "Deep Learning" OR AB "Deep Learning" | 2,883 |
| S4 | MW "Decision tree" OR TI "Decision tree" OR AB "Decision tree" | 2,185 |
| S5 | MW "K-Nearest Neighbor*" OR TI "K-Nearest Neighbor*" OR AB "K-Nearest Neighbor*" | 351 |
| S6 | MW "Support vector machine*" OR TI "Support vector machine*" OR AB "Support vector machine*" | 2,122 |
| S7 | MW "Recurrent neural network*" OR TI "Recurrent neural network*" OR AB "Recurrent neural network*" | 178 |
| S8 | MW "convolutional neural network*" OR TI "convolutional neural network*" OR AB "convolutional neural network*" | 1,177 |
| S9 | MW "Artificial neural network*" OR TI "Artificial neural network*" OR AB "Artificial neural network*" | 1,246 |
| S10 | MW "Naïve Bayes" OR TI "Naïve Bayes" OR AB "Naïve Bayes" | 221 |
| S11 | MW "Naive Bayes" OR TI "Naive Bayes" OR AB "Naive Bayes" | 151 |
| S12 | MW "Fuzzy Logic" OR TI "Fuzzy Logic" OR AB "Fuzzy Logic" | 243 |
| S13 | MW "K-Means" OR TI "K-Means" OR AB "K-Means" | 830 |
| S14 | MW "Random Forest" OR TI "Random Forest" OR AB "Random Forest" | 1,597 |
| S15 | MW "Long Short-Term Memory Networks" OR TI "Long Short-Term Memory Networks" OR AB "Long Short-Term Memory Networks" | 13 |
| S16 | MW "serious gam*" OR TI "serious gam*" OR AB "serious gam*" | 423 |
| S17 | MW "game-based" OR TI "game-based" OR AB "game-based" | 457 |
| S18 | MW "videogam*" OR TI "videogam*" OR AB "videogam*" | 328 |
| S19 | MW "video game*" OR TI "video gam*" OR AB "video gam*" | 5,878 |
| S20 | MW gamified OR TI gamified OR AB gamified | 182 |
| S21 | MW exergam* OR TI exergam* OR AB exergam* | 501 |
| S22 | MW "Applied game*" OR TI "Applied game*" OR AB "Applied game*" | 10 |
| S23 | MW "virtual reality game*" OR TI "virtual reality game*" OR AB "virtual reality game*" | 68 |
| S24 | MW "virtual reality-based game*" OR TI "virtual reality-based game*" OR AB "virtual reality-based game*" | 2 |
| S25 | MW "augmented reality-based game*" OR TI "augmented reality-based game*" OR AB "augmented reality-based game*" | 0 |
| S26 | MW "augmented reality game*" OR TI "augmented reality game*" OR AB "augmented reality game*" | 21 |
| S27 | S1 OR S2 OR S3 OR S4 OR S5 OR S6 OR S7 OR S8 OR S9 OR S10 OR S11 OR S12 OR S13 OR S14 OR S15) | 22,641 |
| S28 | S16 OR S17 OR S18 OR S19 OR S20 OR S21 OR S22 OR S23 OR S24 OR S25 OR S26 | 6,908 |
| S29 | (S16 OR S17 OR S18 OR S19 OR S20 OR S21 OR S22 OR S23 OR S24 OR S25 OR S26) AND (S27 AND S28) | 60 |
| S30 | limit 29 to english language | 59 |

| **Database** | **Query** | **Results** |
| --- | --- | --- |
| **IEEE Xplore** | ("Abstract":"Artificial Intelligence" OR "Abstract":"Machine Learning" OR "Abstract":"Deep Learning" OR "Abstract":"Decision tree" OR "Abstract":"K-Nearest Neighbor" OR "Abstract":"K-Nearest Neighbors" OR "Abstract":"Support vector machine" OR "Abstract":"Support vector machines" OR "Abstract":"Recurrent neural network" OR "Abstract":"convolutional neural network" OR "Abstract":"Artificial neural network" OR "Abstract":"Recurrent neural networks" OR "Abstract":"convolutional neural networks" OR "Abstract":"Artificial neural networks" OR "Abstract":"Naïve Bayes" OR "Abstract":"Naive Bayes" OR "Abstract":"Fuzzy Logic" OR "Abstract":"logistic regression" OR "Abstract":"K-Means" OR "Abstract":"Random Forest" OR "Abstract":"Long Short-Term Memory Networks") AND ("Abstract":"serious game" OR "Abstract":"serious games" OR "Abstract":"game-based" OR "Abstract":"videogame" OR "Abstract":"video game" OR "Abstract":"videogames" OR "Abstract":"video games" OR "Abstract":gamification OR "Abstract":gamified OR "Abstract":exergame OR "Abstract":exergames OR "Abstract":"Applied game*" OR "Abstract":"virtual reality game*") Filters Applied: 2010 - 2022 | 424 |
| **ACM Digital library** | [[Abstract: "serious game"] OR [Abstract: "serious games"] OR [Abstract: "game-based"] OR [Abstract: "videogame"] OR [Abstract: "video game"] OR [Abstract: "videogames"] OR [Abstract: "video games"] OR [Abstract: gamification] OR [Abstract: gamified] OR [Abstract: exergame] OR [Abstract: exergames] OR [Abstract: "applied game*"] OR [Abstract: "virtual reality game*"]] AND [[Abstract: "artificial intelligence"] OR [Abstract: "machine learning"] OR [Abstract: "deep learning"] OR [Abstract: "decision tree"] OR [Abstract: "k-nearest neighbor"] OR [Abstract: "k-nearest neighbors"] OR [Abstract: "support vector machine"] OR [Abstract: "support vector machines"] OR [Abstract: "recurrent neural network"] OR [Abstract: "convolutional neural network"] OR [Abstract: "artificial neural network"] OR [Abstract: "recurrent neural networks"] OR [Abstract: "convolutional neural networks"] OR [Abstract: "artificial neural networks"] OR [Abstract: "naïve bayes"] OR [Abstract: "naive bayes"] OR [Abstract: "fuzzy logic"] OR [Abstract: "logistic regression"] OR [Abstract: "k-means"] OR [Abstract: "random forest"] OR [Abstract: "long short-term memory networks"]] AND [Publication Date: (01/01/2010 TO 12/31/2022)] | 120 |
| **Google Scholar** | ("Artificial Intelligence" OR "Machine Learning" OR "Deep Learning" OR "Support vector machine*" OR "Recurrent neural network*" OR "convolutional neural network*") AND ("serious gam*" OR "game-based" OR exergam* OR gamify* OR "video game*") | 100 |
