# Supplementary material for: Artificial Intelligence–Driven Serious Games in Health Care: Scoping Review
Source: JMIR Serious Games. 2022 Nov 29;10(4):e39840. doi: 10.2196/39840 (PMC9748798; doi:10.2196/39840)
Supplement: Multimedia Appendix 3 [file games_v10i4e39840_app3.docx]

| **Concept** | **Definition** |
| --- | --- |
| **Study Characteristics** |  |
| Author | The first author of the study. |
| Year of publication | The year in which the study was published. |
| Country of publication | The country where the study was published. |
| Type of publication | The venue where the study was published: peer-reviewed journal articles, book chapters, dissertations, or conference proceedings |
| **Serious game characteristics** |  |
| Name of the serious game | The name given for the serious game (e.g., SPARX, Tetris, etc..). |
| Targeted health condition | What is the health condition/disease that the AI-based serious game targeted (depression, anxiety, dyslexia, diabetes, autism, ADHD, etc..)? |
| Therapeutic modality | What is the therapy that the serious game provides? |
| Interface | Is the virtual world inside the game three-dimensions (3D) (like the real world) or two-dimensions (2D) (a top-down scrolling and side-scrolling for the perspective of a simpler world) |
| Number of players | How many players who simultaneously playing the game (Single-player or multiplayer)? |
| Connectivity | Does the game require an internet connection to be played (online or offline)? |
| Serious game genre | What is the type of gameplay (e.g., Simulation, action, Puzzle, Adventure, Strategy, Quiz, Sport, Platformer )? |
| Serious game type | What is the type of serious games?   1. Designed serious games: games that are designed with a “serious” purpose from the beginning. 2. Purpose-shifted serious games: games that were not designed as serious games but are being used for a serious purpose. 3. Modified serious games: games that are similar to purpose-shifted ones, but while purpose-shifted games are left intact, modified ones can differ from the original in terms of gameplay and characters. |
| Game Engine | What is the platform that was used to create and develop the serious game? |
| Platform | The platform in which the serious game is implemented (e.g., mobile, tablet, PC, Console, wearable devices, etc..). |
| Other connected devices | What are the other technologies that were connected to the serious games? |
| **AI** **Characteristics** |  |
| AI algorithm used | What are the AI algorithms/models (e.g., RF, SVM, ANN, CNN, RNN, DNN, k-NN, MLP, DBN, DBM, DPN BN, CRT, DT, LASSO, LR, MFA, MLR, MDL, NB, NN, NSC, RBFN) used in the serious game? |
| Type of data | What is the type of data (e.g., Neuroimaging, biological, radiology images, clinical data, laboratory data, epidemiological data, and demographical data) that were used for developing the algorithm? |
| Sample size | What is the number of participants from which the data was collected? |
| Dataset size | What is the dataset size used for developing the algorithm? |
| Type of validation | What is the approach that was used to validate the developed algorithm (e.g., Training-test split, K-fold cross-validation, Nested Cross-Validation, Leave One Out cross-validation, Apparent validation, external validation)? |
| Performance measures used | What are the measures used to assess the performance of the algorithm (accuracy, sensitivity (recall), specificity, precision, AUC, etc...)? |
