# Supplementary material for: Artificial Intelligence–Driven Serious Games in Health Care: Scoping Review
Source: JMIR Serious Games. 2022 Nov 29;10(4):e39840. doi: 10.2196/39840 (PMC9748798; doi:10.2196/39840)
Supplement: Multimedia Appendix 4 [file games_v10i4e39840_app4.docx]

**Multimedia Appendix 4: Characteristics of each included study**

| Study [Ref] | Year | Country | Publication type |
| --- | --- | --- | --- |
| Alchalabi et al [40] | 2017 | Turkey | Conference paper |
| Alchalabi et al [41] | 2018 | Turkey | Journal article |
| Aljumaili et al [42] | 2019 | Canada | Conference paper |
| Alshurafa et al [43] | 2014 | USA | Journal article |
| Anzulewicz et al [44] | 2016 | Poland | Journal article |
| Ascari et al [45] | 2020 | Brazil | Conference Paper |
| Avola et al [46] | 2019 | Italy | Journal article |
| Baur et al [47] | 2017 | Switzerland | Journal article |
| Burdea et al [48] | 2021 | USA | Journal article |
| Chen et al [49] | 2018 | Taiwan | Conference Paper |
| Chiu et al [50] | 2018 | Taiwan | Journal article |
| Esfahlani et al [51] | 2019 | UK | Journal article |
| Farahanipad et al [52] | 2020 | USA | Conference paper |
| Frutos-Pascual et al [53] | 2015 | Spain | Journal article |
| Fuertes et al [54] | 2017 | Ecuador | Conference paper |
| Garcia-Agundez et al [55] | 2019 | Germany | Journal article |
| Gielis et al [56] | 2021 | Belgium | Journal article |
| Heller et al [57] | 2013 | USA | Journal article |
| Huang et al [58] | 2017 | Australia | Journal article |
| Jung et al [59] | 2018 | South Korea | Journal article |
| Kariyawasam et al [60] | 2019 | Sri Lanka | Conference paper |
| Liu et al [61] | 2014 | Singapore | Conference paper |
| Macintosh et al [62] | 2021 | Canada | Journal article |
| Mansart et al [63] | 2015 | Thailand | Conference paper |
| Marín-Morales et al [64] | 2021 | Spain | Conference paper |
| Mavandadi et al [65] | 2012 | USA | Journal article |
| Morando et al [66] | 2019 | Italy | Conference paper |
| Munoz et al [67] | 2014 | Columbia | Journal article |
| Najeeb et al [68] | 2020 | Sri Lanka | Conference paper |
| Nasri et al [69] | 2020 | Spain | Journal article |
| Oliver et al [70] | 2018 | Spain | Journal article |
| Ortiz-Catalan et al [71] | 2016 | Sweden, Slovenia | Journal article |
| Perez-Muñoz et al [72] | 2018 | Ecuador | Conference paper |
| Postolache et al [73] | 2015 | Portugal | Conference paper |
| Puzenat et al [74] | 2010 | France | Conference paper |
| Rohani et al [75] | 2014 | Denmark | Journal article |
| Sakoda et al [76] | 2020 | Japan | Conference paper |
| Sourial et al [77] | 2016 | Egypt | Conference paper |
| Valladares-Rodriguez et al [78] | 2018 | Spain | Journal article |
| van Diest et al [79] | 2015 | Netherlands | Journal article |
| Varga et al [80] | 2021 | Romania | Journal article |
| Vonstad et al [81] | 2021 | Norway | Journal article |
| Wang et al [82] | 2018 | China | Conference paper |
| Yeh et al [83] | 2014 | Taiwan | Journal article |
| Zainal et al [84] | 2019 | Malaysia | Conference paper |
| Zhang et al [85] | 2017 | Singapore | Conference paper |
