# Supplementary material for: Artificial Intelligence–Driven Serious Games in Health Care: Scoping Review
Source: JMIR Serious Games. 2022 Nov 29;10(4):e39840. doi: 10.2196/39840 (PMC9748798; doi:10.2196/39840)
Supplement: Multimedia Appendix 5 [file games_v10i4e39840_app5.docx]

**Multimedia Appendix 5: Characteristics of the serious games in the included studies**

| Study | Name of the game | Targeted health condition | Purpose | Therapeutic modality | 2D or 3D | Players | Web-based or Offline | Genre | Type | Game Engine | Platform | Other connected devices |
| --- | --- | --- | --- | --- | --- | --- | --- | --- | --- | --- | --- | --- |
| Alchalabi et al [40] | FOCUS | ADHD^a^ | Rehabilitation | Cognitive training | 3D | Single | Offline | Role-playing | Designed | Unity | PC | Wearable sensors |
| Alchalabi et al [41] | FOCUS | ADHD | Rehabilitation | Cognitive training | 3D | Single | Offline | Role-playing | Designed | Unity | PC | Wearable sensors |
| Aljumaili et al [42] | WarCAT | MCI^b^ | Detection | N/A^c^ | 2D | Single | Offline | Puzzle | Designed | Android | Mobile devices | N/A |
| Alshurafa et al [43] | FreedroidRPG | Any health condition | Health and wellness | N/A | 2D | Single | Offline | Role-playing | Modified | Linux | Treadmill | Wearable sensors |
| Anzulewicz et al [44] | Sharing, Creativity | Autism | Detection | N/A | 2D | Single | Offline | Role-playing and painting | Modified | iOS | Mobile devices | N/A |
| Ascari et al [45] | PGCA | Motor impairment and speech impairment | Rehabilitation | Exercise | 2D | Single | Offline | Platformer | Designed | Windows | PC | Web camera |
| Avola et al [46] | NR^d^ | Motor impairment | Rehabilitation | Exercise | 3D | Single | Offline | Role-playing | Designed | Unity | VR^e^ headset | Nonwearable sensors, wearable devices, and wearable sensors |
| Baur et al [47] | Air hockey | Motor impairment | Rehabilitation | Exercise | 2D | Multi | Offline | Sports | Designed | NR | PC | Robotic device |
| Burdea et al [48] | BrightBrainer Grasp | Motor impairment | Rehabilitation | Exercise | 2D and 3D | Single | Offline | Sports, puzzle, and musical | Designed | Windows | PC | Wearable devices and wearable sensors |
| Chen et al [49] | Robot game, Shot game, Study game, and Firework game | Back and neck pain | Rehabilitation and health and wellness | Exercise | 2D | Single | Offline | Platformer | Designed | Linux 9 | Mobile devices | Wearable sensors and tiny PC |
| Chiu et al [50] | NR | Motor impairment | Rehabilitation | Exercise | 2D | Single | Offline | Painting | Designed | NR | Mobile devices | Nonwearable sensors |
| Esfahlani et al [51] | NR | Motor impairment | Rehabilitation | Exercise | 3D | Single | Offline | Role-playing | Designed | Unity | PC | Nonwearable sensors and wearable sensors |
| Farahanipad et al [52] | HandReha | Motor impairment | Rehabilitation | Exercise | 3D | Single | Offline | Action | Designed | Godot | PC | Web camera |
| Frutos-Pascual et al [53] | NR | ADHD | Rehabilitation | Cognitive training | 2D | Single | Offline | Puzzle | Designed | Ubuntu | PC | Nonwearable sensors |
| Fuertes et al [54] | TicTacToe | Any health condition | Education | N/A | 3D | Single | Offline | Puzzle | Designed | Unity | PC | Microphone and web camera |
| Garcia-Agundez et al [55] | VR-Flight | Cybersickness | Detection | N/A | 3D | Single | Offline | Platformer | Designed | Unity | PC | Wearable devices, wearable sensors, web camera, and controller |
| Gielis et al [56] | Klondike Solitaire | MCI | Detection | N/A | 2D | Single | Offline | Puzzle | Modified | NR | Mobile devices | N/A |
| Heller et al [57] | Groundskeeper | ADHD | Detection | N/A | 2D | Single | Offline | Role-playing and painting | Designed | C++ framework | Sifteo cubes | N/A |
| Huang et al [58] | SpaceWar, Flying bird, Spaceship, Transferring supermarket, and Transferring kitchen | Motor impairment | Rehabilitation | Exercise | 2D and 3D | Single | Offline | Role-playing and action | Designed | Unity | PC | Nonwearable sensors, robotic device, and wearable devices |
| Jung et al [59] | Neuro-World | Cognitive impairment | Detection | N/A | 3D | Single | Offline | Puzzle | Designed | Android | Mobile devices | N/A |
| Kariyawasam et al [60] | Pubudu | Learning disabilities | Rehabilitation | Cognitive training | 2D | Single | Offline | Quiz | Designed | Cocos2dx | Mobile devices | N/A |
| Liu et al [61] | Pumpkin Garden | Parkinson disease | Detection | N/A | 2D | Single | Offline | Role-playing | Designed | iOS | Mobile devices | N/A |
| Macintosh et al [62] | Dashy Square | Cerebral palsy | Rehabilitation | Biofeedback | 2D | Single | Offline | Platformer | Modified | MATLAB | PC | Wearable sensors |
| Mansart et al [63] | Go Run Go | Any health condition | Health and wellness | N/A | 2D | Single | Web-based | Sports | Designed | Android | Mobile devices | N/A |
| Marín-Morales et al [64] | Expanse | Neuropsychological disorders | Detection | N/A | 3D | Single | Offline | Adventure | Designed | Unity | VR headset | Wearable devices |
| Mavandadi et al [65] | NR | Malaria | Detection | N/A | 2D | Single | Web-based | Puzzle | Designed | Adobe Flash and Android | Multi | N/A |
| Morando et al [66] | ReMoVES | Motor impairment | Rehabilitation | Exercise | 2D | Single | Web-based | Role-playing | Designed | php and react frameworks | PC | Nonwearable sensors |
| Munoz et al [67] | NR | Motor impairment | Rehabilitation | Biofeedback | 3D | Single | Web-based | Role-playing | Designed | Unity | PC | Wearable sensors |
| Najeeb et al [68] | Aliza | Autism | Education | N/A | 2D | Single | Offline | Puzzle and quiz | Designed | Unity | Single-board computer | Microphone, monitor, speakers, and web camera |
| Nasri et al [69] | NR | Motor impairment | Rehabilitation | Biofeedback | 3D | Single | Offline | Platformer | Designed | Unity | PC | Wearable sensors |
| Oliver et al [70] | NR | Cognitive impairment | Rehabilitation | Cognitive training and exercise | 2D | Single | Web-based | Puzzle | Designed | NR | PC | Nonwearable sensors and wearable sensors |
| Ortiz-Catalan et al [71] | Neuromotus | Phantom limb pain | Rehabilitation | Biofeedback | 3D | Single | Offline | Sports | Designed | NR | PC | Wearable sensors and web camera |
| Perez-Muñoz et al [72] | FarmFunTime, Find the Food, Color it, Dodge it, Catch the Balloons, and Imitate it | Motor impairment | Rehabilitation | Exercise | 2D and 3D | Single | Web-based | Painting, platformer, puzzle, and simulation | Designed | Java framework | PC | Nonwearable sensors |
| Postolache et al [73] | Therasoup and Apples Harvesting | Motor impairment | Rehabilitation | Exercise | 3D | Single | Web-based | Role-playing | Designed | Unity and Android | PC | Nonwearable sensors and smartphone |
| Puzenat et al [74] | SuperTuxKart, Shadows, Super balls, Colors, and Super maze | Any health condition | Prediction | N/A | 2D and 3D | Single | Offline | Puzzle, quiz, and platformer | Designed | Java framework, Linux, and Windows | PC | N/A |
| Rohani et al [75] | Anispell and T-Search | ADHD | Rehabilitation | Cognitive training | 3D | Single | Offline | Role-playing | Designed | Unity | PC | Nonwearable sensors and wearable sensors |
| Sakoda et al [76] | Ski Exergame | Locomotive syndrome | Health and wellness | N/A | 3D | Single | Offline | Sports | Designed | Unity | VR headset | Nonwearable sensors and wearable devices |
| Sourial et al [77] | NR | Motor impairment | Rehabilitation | Exercise | 3D | Single | Web-based | Simulation | Designed | Unity | PC | Nonwearable sensors, web camera, and microphone |
| Valladares-Rodriguez et al [78] | Panoramix | MCI and Alzheimer disease | Detection | N/A | 2D | Single | Offline | Quiz | Designed | Unity | PC | Controllers |
| van Diest et al [79] | Ice-skating | Postural imbalance | Rehabilitation | Exercise | 3D | Single | Offline | Sports | Designed | NR | PC | Nonwearable sensors |
| Varga et al [80] | NR | Rheumatoid arthritis | Rehabilitation | Exercise | 3D | Single | Offline | Platformer | Designed | Unity | PC | Nonwearable sensors |
| Vonstad et al [81] | NR | Motor impairment | Health and wellness | N/A | 3D | Single | Offline | Platformer | Designed | Unity | PC | Nonwearable sensors |
| Wang et al [82] | NR | Motor impairment | Rehabilitation | Biofeedback | 3D | Single | Offline | Role-playing | Designed | Unity | PC | Robotic device and wearable sensors |
| Yeh et al [83] | NR | Vestibular dysfunction | Rehabilitation | Exercise | 3D | Single | Offline | Quiz and sport | Designed | NR | VR headset | Nonwearable sensors and wearable devices |
| Zainal et al [84] | Izzy the Bee and Atlantis | Motor impairment | Rehabilitation | Exercise | 3D | Single | Offline | Platformer | Designed | C++ framework | PC | Nonwearable sensors |
| Zhang et al [85] | NR | Motor impairment | Rehabilitation | Exercise | 3D | Single | Offline | Sports | Designed | NR | PC | Nonwearable sensors |

^a^ADHD: attention deficit hyperactivity disorder.

^b^MCI: mild cognitive disorder.

^c^N/A: not applicable.

^d^NR: not reported.

^e^VR: virtual reality.
