# Supplementary material for: Artificial Intelligence–Driven Serious Games in Health Care: Scoping Review
Source: JMIR Serious Games. 2022 Nov 29;10(4):e39840. doi: 10.2196/39840 (PMC9748798; doi:10.2196/39840)
Supplement: Multimedia Appendix 6 [file games_v10i4e39840_app6.docx]

**Multimedia Appendix 6: Characteristics of artificial intelligence techniques leveraged by serious games in the included studies.**

| Study | Task type | Algorithms | Purpose of artificial intelligence | Data type | Sample size | Dataset size | Type of validation | Performance measures |
| --- | --- | --- | --- | --- | --- | --- | --- | --- |
| Alchalabi et al [40] | Classification | K-NN^a^ and SVM^b^ | Detection of disease | Biosignals | 5 | 4015 | K-fold cross-validation | ACC^c^, SE^d^, PR^e^, and *F*_1_-score |
| Alchalabi et al [41] | Classification | SVM | Detection of disease | Biosignals | 5 | 53,790 | K-fold cross-validation | ACC |
| Aljumaili et al [42] | Classification | CNN^f^ and Q learning | Detection of disease | Gameplay data | 150 | 55,000 | Training-test split | ACC |
| Alshurafa et al [43] | Classification and clustering | DT^g^, Gaussian mixture model, K-MC^h^, K-NN, and SVM | Classification of activity | Biosignals | 12 | 510 | Leave-one-out cross-validation | SE, PR, and *F*_1_-score |
| Anzulewicz et al [44] | Classification | ET^i^, random forest RF^j^, and regularized greedy forest | Detection of disease | Demographic data and kinematic data | 82 | 262 | K-fold cross-validation | AUC^k^, SE, and SP^l^ |
| Ascari et al [45] | Classification | CNN and SVM | Recognition of gestures | Kinematic data | 3 | NR | K-fold cross-validation and leave-one-out cross-validation | ACC |
| Avola et al [46] | Classification | Gated recurrent unit recurrent neural network | Evaluation of user’s performance | Kinematic data | 24 | 3600 | NR | ACC |
| Baur et al [47] | Classification | NR^m^ | Adaptation of difficulty level | Gameplay data | 6 | NR | Moving-window cross-validation | NR |
| Burdea et al [48] | Classification | NR | Adaptation of difficulty level | Gameplay data and kinematic data | NR | NR | NR | NR |
| Chen et al [49] | Classification | CNN and fully connected neural network | Evaluation of user’s performance | Kinematic data | NR | 36 | Training-test split | ACC |
| Chiu et al [50] | Classification and regression | FL^n^ | Evaluation of user’s performance | Kinematic data | NR | NR | NR | NR |
| Esfahlani et al [51] | Classification | ANN^o^, FL, and inverse kinematics | Adaptation of difficulty level, evaluation of user’s performance, and supporting users to play | Kinematic data | 52 | 3658 | Training-test split | ACC |
| Farahanipad et al [52] | Classification | CNN | Recognition of gestures | Kinematic data | 12 | 7405 | Training-test split | ACC |
| Frutos-Pascual et al [53] | Classification | AB^p^, DT, extremely randomized trees, and RF | Evaluation of user’s performance | Gameplay data and kinematic data | 32 | NR | K-fold cross-validation | ACC |
| Fuertes et al [54] | Classification | NR | Recognition of gestures and recognition of voice | Kinematic data and speech data | 120 | NR | NR | NR |
| Garcia-Agundez et al [55] | Classification | K-NN, neural network, and SVM | Detection of disease | Biosignals and gameplay data | 66 | NR | Training-test split | ACC |
| Gielis et al [56] | Classification | ET, gradient boosting, and nu-SVC^q^ | Detection of disease | Gameplay data | 46 | 138 | K-fold cross-validation | ACC, SE, SP, PR, NPV^r^, *F*_1_-score, and AUC |
| Heller et al [57] | Classification | AB, J48, JRip, and RF | Detection of disease | Gameplay data | 52 | 795,600 | Training-test split | ACC, SE, SP, PR, NPV, and *F*_1_-score |
| Huang et al [58] | Regression | Reinforcement learning neural network | Adaptation of difficulty level | Kinematic data | 8 | NR | NR | NR |
| Jung et al [59] | Regression | RF | Detection of disease | Gameplay data | 12 | 864 | Leave-one-out cross-validation | Root mean square error and normalized root mean square error |
| Kariyawasam et al [60] | Classification | CNN, DT, K-NN, LR^s^, NB^t^, RF, and SVM | Detection of disease | Gameplay data | NR | NR | NR | ACC |
| Liu et al [61] | Regression | FL | Detection of disease | Gameplay data | 20 | NR | NR | NR |
| Macintosh et al [62] | Classification | K-NN, RF, and SVM | Recognition of biosignals | Biosignals and gameplay data | 19 | 51,509 | K-fold cross-validation | SE, *F*_1_-score, and Mathew correlation coefficient |
| Mansart et al [63] | Classification | DT | Classification of activity | Kinematic data | 10 | 19,615 | Training-test split | ACC, SE, PR, and *F*_1_-score |
| Marín-Morales et al [64] | Classification | DT, elastic-net regularized generalized linear model, K-NN, NB, and SVM | Detection of disease | Gameplay data | 60 | 920 | K-fold cross-validation | ACC, SE, SP, and kappa |
| Mavandadi et al [65] | Classification | AB | Detection of disease | Laboratory data | NR | 6321 | Training-test split | ACC, SE, SP, PR, and NPV |
| Morando et al [66] | Classification | SVM | Evaluation of user’s performance | Kinematic data | NR | 389 | K-fold cross-validation | ACC |
| Munoz et al [67] | Classification | Linear discriminant analysis, nu-SVC, and SVM | Recognition of biosignals | Biosignals | 8 | NR | K-fold cross-validation | ACC |
| Najeeb et al [68] | Classification | CNN | Evaluation of user’s performance | Gameplay data | 100 | 111,829 | Training-test split | ACC |
| Nasri et al [69] | Classification | Gated recurrent unit conventional neural network | Recognition of biosignals | Biosignals | 15 | 18,500 | Leave-one-out cross-validation | ACC |
| Oliver et al [70] | Classification | FL | Adaptation of difficulty level and supporting users to play | Biosignals and gameplay data | NR | NR | NR | NR |
| Ortiz-Catalan et al [71] | Classification | NR | Recognition of biosignals | Biosignals | 14 | NR | NR | NR |
| Perez-Muñoz et al [72] | Classification | FL | Adaptation of difficulty level | Clinical data and gameplay data | 5 | NR | NR | NR |
| Postolache et al [73] | Classification | ANN | Recognition of gestures | Kinematic data | NR | NR | NR | NR |
| Puzenat et al [74] | Classification | ANN | Prediction of user’s characteristics | Demographic data and gameplay data | 40 | NR | Leave-one-out cross-validation | ACC |
| Rohani et al [75] | Classification | SVM | Evaluation of user’s performance | Biosignals | 5 | NR | K-fold cross-validation and training-test split | AUC, SE, and SP |
| Sakoda et al [76] | Classification | ANN | Adaptation of difficulty level | Gameplay data and kinematic data | 5 | NR | NR | NR |
| Sourial et al [77] | Classification | NR | Evaluation of user’s performance, recognition of gestures, recognition of voice, and supporting users to play | Kinematic data and speech data | 129 | 600 | NR | ACC |
| Valladares-Rodriguez et al [78] | Classification | Classification and regression tree, LR, and SVM | Detection of disease | Demographic data and gameplay data | 16 | 89 | Leave-one-out cross-validation | ACC and *F*_1_-score |
| van Diest et al [79] | Classification | ANN and K-NN | Recognition of gestures | Kinematic data | 40 | 113,400 | Training-test split | ACC |
| Varga et al [80] | Classification | NR | Recognition of gestures | Gameplay data and kinematic data | NR | NR | Training-test split | ACC, SE, PR, and *F*_1_-score |
| Vonstad et al [81] | Classification | ANN, K-NN, RF, and SVM | Evaluation of user’s performance | Kinematic data | 12 | 2821 | K-fold cross-validation and leave-one-out cross-validation | SE and *F*_1_-score |
| Wang et al [82] | Classification | ANN | Recognition of biosignals | Biosignals | NR | 100 | Training-test split | NR |
| Yeh et al [83] | Classification | SVM | Evaluation of user’s performance | Kinematic data | 84 | NR | K-fold cross-validation | ACC |
| Zainal et al [84] | Clustering | K-MC | Evaluation of user’s performance | Gameplay data and kinematic data | 19 | NR | NR | NR |
| Zhang et al [85] | Regression | FL | Evaluation of user’s performance | Gameplay data and kinematic data | 5 | NR | NR | NR |

^a^K-NN: k-nearest neighbors.

^b^SVM: support vector machine.

^c^ACC: accuracy.

^d^SE: sensitivity.

^e^PR: precision.

^f^CNN: convolutional neural network.

^g^DT: decision tree.

^h^K-MC: k-means clustering.

^i^ET: ExtraTrees.

^j^RF: random forest.

^k^AUC: area under the curve.

^l^SP: specificity.

^m^NR: not reported.

^n^FL: fuzzy logic.

^o^ANN: artificial neural network.

^p^AB: AdaBoost.

^q^nu-SVC: nu-support vector classifier.

^r^NPV: negative predictive value.

^s^LR: logistic regression.

^t^NB: Naïve Bayes.
